# Robotic Micropipette Aspiration for Multiple Cells

**DOI:** 10.3390/mi10050348

**Published:** 2019-05-27

**Authors:** Yaowei Liu, Maosheng Cui, Jingjing Huang, Mingzhu Sun, Xin Zhao, Qili Zhao

**Affiliations:** 1 Institute of Robotics and Automatic Information System and the Tianjin Key Laboratory of Intelligent Robotics, Nankai University, Tianjin 300071, China; liuyaowei@mail.nankai.edu.cn (Y.L.); sunmz@nankai.edu.cn (M.S.); zhaoxin@nankai.edu.cn (X.Z.); 2 Institute of Animal Sciences, Tianjin 300112, China; tjxmcui2014@126.com (M.C.); hjingjing00@163.com (J.H.)

**Keywords:** micropipette aspiration, robotic batch cell manipulation, Young’s modulus measurement, cell transportation, cell detection, force analysis

## Abstract

As there are significant variations of cell elasticity among individual cells, measuring the elasticity of batch cells is required for obtaining statistical results of cell elasticity. At present, the micropipette aspiration (MA) technique is the most widely used cell elasticity measurement method. Due to a lack of effective cell storage and delivery methods, the existing manual and robotic MA methods are only capable of measuring a single cell at a time, making the MA of batch cells low efficiency. To address this problem, we developed a robotic MA system capable of storing multiple cells with a feeder micropipette (FM), picking up cells one-by-one to measure their elasticity with a measurement micropipette (MM). This system involved the following key techniques: Maximum permissible tilt angle of MM and FM determination, automated cell adhesion detection and cell adhesion break, and automated cell aspiration. The experimental results demonstrated that our system was able to continuously measure more than 20 cells with a manipulation speed quadrupled in comparison to existing methods. With the batch cell measurement ability, cell elasticity of pig ovum cultured in different environmental conditions was measured to find optimized culturing protocols for oocyte maturation.

## 1. Introduction

The mechanical properties of living cells play a key role in cell physiology and pathology [1,2,3,4,5] and can help gain insights in cell structures and functions [6].The oocyte elasticity has especially been found to play a vital role in many physiological processes of animal or cells. It has been demonstrated that the oocyte Young’s modulus can be useful for investigating disease mechanisms and mature process, since the biomechanical properties of the pathological/immature cells can differ from healthy/mature ones. For example, the oocyte Young’s modulus of the mature oocyte is smaller than that of the immature oocyte [7], making cell Young’s modulus a criterion for cell maturation. The oocyte Young’s modulus in mice would change as the mouse became fatter [8]. The oocyte Young’s modulus would become smaller as the mouse aged [9]. The oocyte Young’s modulus would become bigger after fertilization [10]. Thus, the measurement of cell elasticity may provide an effective tool to explore the mechanism of above cell/animal physiological processes.

As there are significant variations of cell Young’s modulus among individual cells even cultured in the same condition, measuring the Young’s modulus of batch cells is requisite for obtaining the statistical results of cell Young’s modulus. The cells’ Young’s modulus may vary with time when cells stay out of the incubator environment for a long time due to cells’ high sensitivity to the culture environment, such as oxygen level [11], temperature [12] and osmolarity [13]. Thus, it is important to measure the Young’s modulus of batch cells in a relatively short time in order to provide a precise evaluation of cell elasticity.

To date, several techniques have been developed to measure the mechanical properties of cells, such as microfluidic techniques [14,15,16], atomic force microscopy (AFM) [17,18,19], optical tweezers techniques [20,21], magnetic tweezers techniques [22,23], and micropipette aspiration (MA) [24,25,26,27,28]. Among these methods, the microfluidic techniques and the micropipette aspiration (MA) may be appropriate for batch Young’s modulus measurement. However, the batch cell measurement by the microfluidic techniques needs to design a specific device for each kind of cell, which limits the application range of this technique. In comparison, the MA method, using a micropipette to aspirate the cell and measure its elasticity, is the most widely used method because of its fewer device requirements, larger suction pressure range, and limited damage to the oocyte. The conventional MA measuring process can be divided into two steps: Cell search and cell measurement. As the cells were usually randomly scattered in the Petri dish, the operators need to search for the target oocyte in a relatively large area every time, which costs a lot of time and significantly reduces the final MA manipulation efficiency. Reference [6] introduced an automated MA system which could automate the cell measurement step. However, that system still needs to search for the target cell each time, making it inappropriate for measure batch cells in a short time. Thus, a MA system for manipulation of batch cells in a short time is still desired for biological applications.

**Figure 1:**
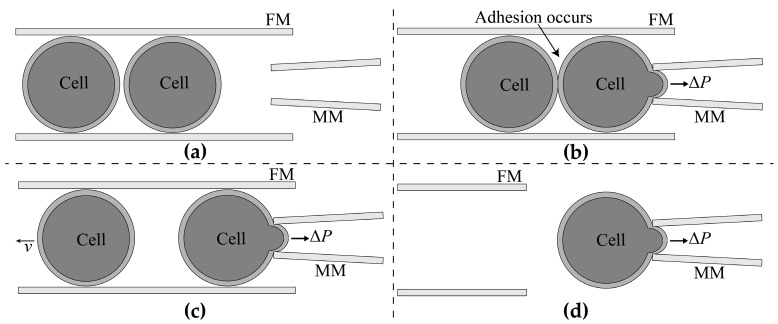
The operation process of new micropipette aspiration (MA) process for batch cells. (**a**) Insert measuring micropipette (MM) into the feeder micropipette (FM). (**b**) Deliver the cells to the microscopic field and hold the target cell by the MM. (**c**) Draw back the adhesive cells with the calculated negative pressure. (**d**) Withdraw the FM and measure the Young’s modulus of the cell by MM.

If the cells can be stored in order instead of being randomly distributed before manipulation, the cell search time can be eliminated, and the fast batch operation of cells can be achieved. Mattos firstly introduced a transportation pipette to store and deliver blastocysts in the injection experiment [29]. The cells are basically linearly distributed in the micropipette as the transportation pipette has a size similar to the cell. The cells were spit out, and the cell searching step could be eliminated. However, in the process of cell delivery, the adhesion between the cells usually happens due to the non-linearity of fluid velocity in the transportation micropipette. In that case, more than one cell is spit out, and it is a challenge for the subsequent cell aspiration, so it is very important to find a way to separate the adhesive cells during cell delivery and finally realize one-by-one cell delivery for MA of batch cells. In order to realize one-by-one cell delivery, we utilized a thinner measurement micropipette to insert into the thicker transportation micropipette to pick up the target cell, then exerted appropriate aspiration force in the transportation micropipette to pull back the adhesive cells. The process involved three key techniques: Determination of the maximum permissible tilt angle of the measurement micropipette (MM) and feeder micropipette (FM) to ensure that the MM can insert into the FM, adhesion detection to judge if the target cell is adhered to other cells, and separation of the adhesive cells using calculated aspiration pressure in FM if adhesion occurs. Figure 1 briefly describes the overall system. Firstly, insert the MM into the FM automatically (Figure 1a). Secondly, deliver the cells to the microscopic field by applying positive pressure in the FM, and hold the target cell by the MM. Thirdly, draw back the adhesive cells if the adhesion occurred. Finally, withdraw the FM and measure the Young’s modulus of the cell by MM.

In this paper, we developed a robotic MA system capable of storing and delivering multiple cells with the FM and picking up one cell at each time with the MM. Firstly, we determined the maximum permissible tilt angle of MM and FM (17°). Secondly, we detected the adhesion between the target cell and the other cells. Thirdly, we calculated the required aspiration pressure to break the adhesion between the adhesive cells (9600 Pa). Finally, we measured the Young’s modulus of the porcine oocytes by the new batch method (*n* = 15) and compared the obtained results with those by traditional MA method (*n* = 15). The experimental results demonstrated that our system was able to continuously operate more than 20 cells one by one, and the average manipulation speed for each cell was quadrupled in comparison to existing methods (2 cell/min vs 0.5 cell/min) (*n* = 22). We measured the Young’s modulus of the porcine oocytes cultured in different environments by this new batch method (*n* = 52). The experimental results showed that the zona pellucida (ZP) Young’s modulus distribution of pig ovum cultured in four different environmental conditions was significantly different, and better culturing protocols for cell maturing could be found.

The rest of the paper is organized as follows: Section 2.1 introduces the key techniques for the batch measurement. The design of the robotic batch measuring process is listed in Section 2.2. The experiments and the results are presented in Section 3, and conclusions are given in Section 4.

## 2. Materials and Methods

### 2.1. Key Techniques

#### 2.1.1. Maximum Permissible Tilt Angle Determination for Measuring Micropipette (MM) and Feeder Micropipette (FM)

**Figure 2:**
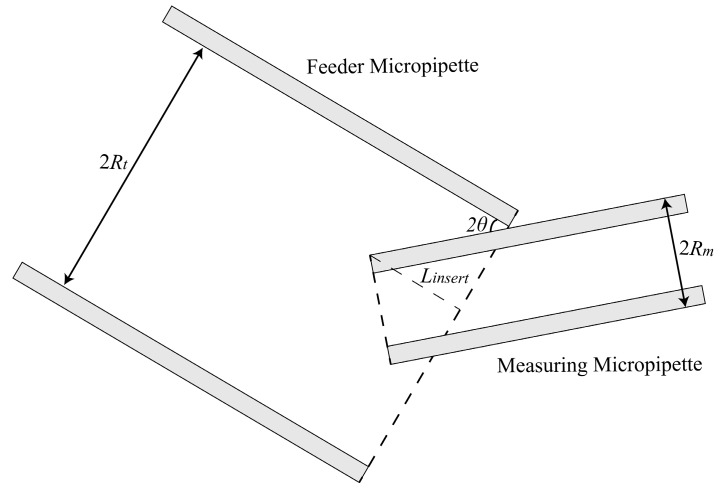
Schematic: The tilted measuring micropipette (MM) is just able to insert into the tilted feeder micropipette (FM).

**Figure 3:**
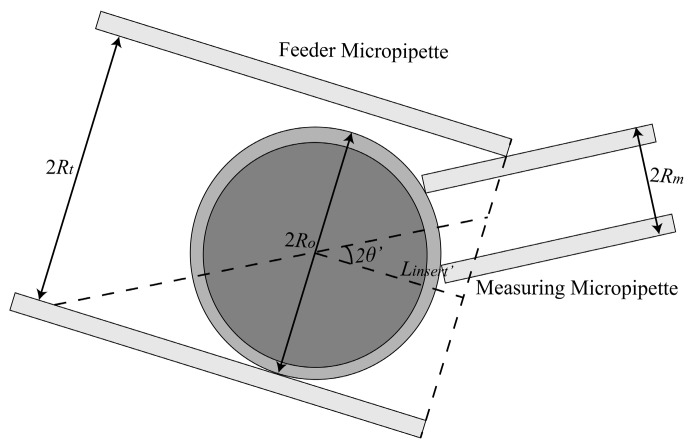
Schematic: The cell almost cannot be aspirated by the tilted measuring micropipette (MM).

To insert the measuring micropipette (MM) into the feeder micropipette (FM) by a certain distance to hold the delivered cell, the tips of two micropipettes are required to be basically horizontally mounted rather than have too large a tilt angle between the tip and horizontal plane. To get the range of required tilt angle, we analyzed the maximum tile angle of the two micropipettes, allowing the MM to insert into the FM (Figure 2) by a certain distance and hold the cell in FM (Figure 3).

As shown in Figure 1, when the MM and the FM have a tilt angle *θ* with the horizontal plane, the relationship between insert length and the tilt angle is obtained as

(1) $$L_{insert}=\left(R_{t}-R_{m}\right)\cot2\theta$$

where *R_t_* is the inner radius of FM, and *R_m_* is the outer radius of the MM.

Then, the biggest degree is determined if the MM is able to insert into the FM by a desired distance according to Equation (1).

Further, we should also consider whether the tilted MM is able to hold the cells in FM. We analyzed the situation that the cell is just able to be held by the MM. As shown in Figure 3, when the MM and the FM are both tilted by the degree of *θ*′, the distance between the oocyte center and the FM opening is *L_insert_*′. According to geometrical relationship, the relationship between *L_insert_*′ and *θ*′ can be obtained as

(2) $$R_{O}+L_{insert}{}^{\prime }\tan2\theta^{\prime }+\frac{R_{m}}{\cos2\theta^{\prime }}=2R_{t}$$

where *R_O_* is the radius of the oocyte.

Based on the above analysis, the biggest tilt angle of FM and MM that allows the MM to hold an oocyte in FM is obtained. In this paper, the inner radius of feeder micropipette *R_t_* is about 100 μm, the outer radius of measuring micropipette *R_m_* is about 20 μm, the radius of the oocyte *R_O_* is about 75–80 μm, and the distance between the oocyte center and the FM opening *L_insert_*’ is set as 120 μm. According to Equation (1) and Equation (2), the biggest tilt angle under this condition is calculated as 17.54°–18.73°. In order to ensure the success of inserting MM into FM, the biggest degree is set as 17° in this paper.

**Figure 4:**
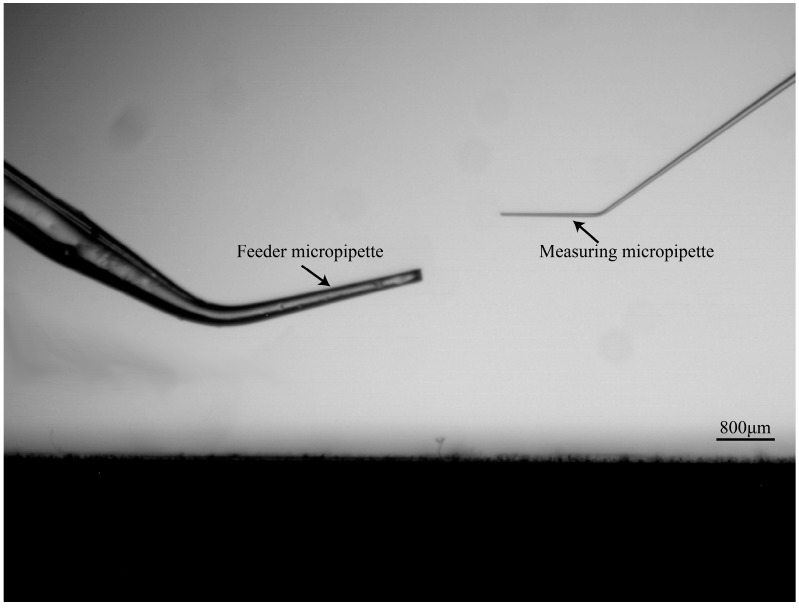
The FM was tilted to 12.3°, and the MM was horizontal.

Further, we developed an imaging processing method to calculate the tilt angle of the above two micropipettes before MA experiment to judge whether the MM is able to insert into the FM to hold the cell. As the used two micropipettes are tubes with basically constant radiuses along their lengths, their widths in microscopy image will basically keep constant if they are horizontally mounted. When the micropipette is mounted with a tilt angle, the defocused state of the micropipette varies along its length direction. If the tip is focused, the blurriness increase resulting from the defocused distance variation will cause an increase of the micropipette width along its length direction in the microscopy image. Based on above analysis, the width variation speed along micropipette length direction is utilized to estimate its tilt angle. We detected the width change speed when the MM and FM were horizontal and tilted with four different degrees (FM: 12.0°, 12.2°, 12.3°, 13.5°; MM: 4.2°, 4.8°, 4.9°, 5.9°). Figure 4 shows the side view picture of a mounted FM tilted by 12.3° and a MM horizontally mounted.

**Figure 5:**
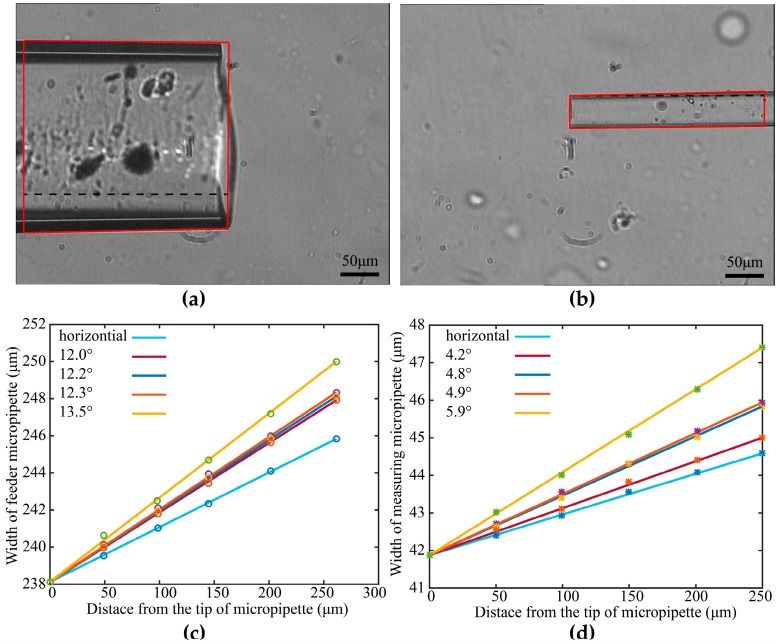
Tilt angle detection results. (**a**) A tilted feeder micropipette (FM). (**b**) A tilted measuring micropipette (MM). The red frames in (**a**,**b**) are the detected edges of the FM and MM. The width changes due to the defocusing along the length direction of the (**c**) feeder micropipette and (**d**) measuring micropipette.

The tips of the two micropipettes were first autofocused according to [30], and the width variation along their length direction was measured using image processing. Figure 5a–d shows the obtained width variation with respect to the distance to the tip of micropipette (along length direction). It can be found that linear relationships between them exist for both FM and MM, no matter the horizontal situation or titled situation. Based on the calculated biggest degree (17°), we had the biggest slope ratio for the FM and the MM. As the slope ratios of horizontal FM and MM are 0.011 and 0.028, the suitable slope ratio of FM and MM for this experiment are 0.011–0.131 and 0.029–0.106, respectively. The two micropipettes were remounted until their detected tilt angles were smaller than the maximum permissible angle.

#### 2.1.2. Adhesion Detection

**Figure 6:**
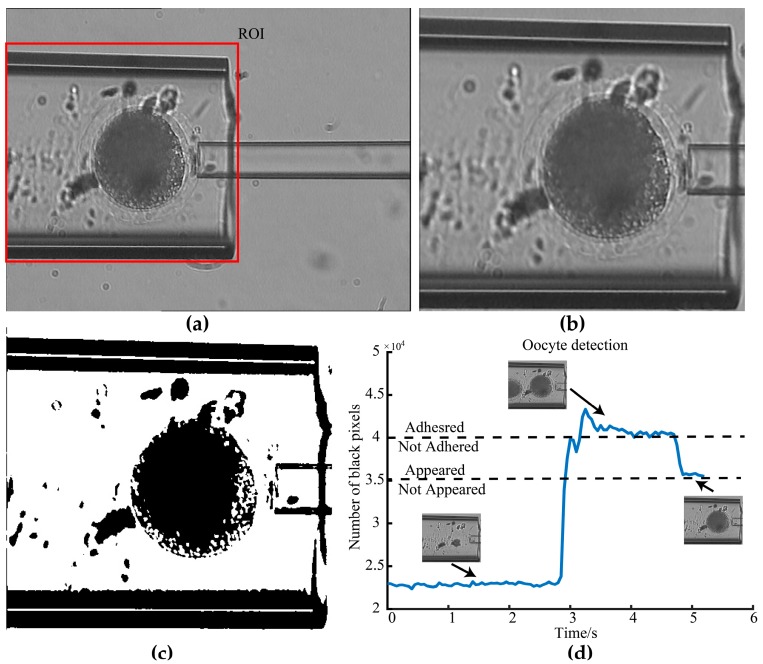
The process of oocyte detection. (**a**) The region of interest (ROI) selection. (**b**) The image after morphological opening. (**c**) The binary image. (**d**) The number of black pixels in the binary image in ROI during the delivery process.

After the two micropipettes were focused and horizontally mounted as mentioned above, the MM was inserted into the FM by 45 μm using previously developed visual back control [31] method. Then, the oocyte was delivered to the microscopic field by giving a positive pressure in the FM. In this paper, the positive pressure was set as 10 kPa, which is experimentally determined for pig oocytes and may be varied when operating cells with other sizes. To hold the cell automatically, we need to detect if the oocyte appears in the microscopic field (see Video S1 for the oocyte detection during the delivery process). Firstly, the region of interest (ROI) was obtained according to detected FM contour using the method in [32], as shown in Figure 6a. Secondly, morphological opening was used to remove the noise of small particles in the ROI, as shown in Figure 6b. Then, the binary image was obtained by using Otsu’s adaptive threshold algorithm, as shown in Figure 6c.

Further, the number of black pixels in the binary image in ROI during the delivery process was counted, as shown in Figure 6d. When the oocyte arrived in the ROI, the cytoplasm significantly increased black pixel numbers in the binary image. For the pig oocyte, when the number of black pixels exceeded 3.5 × 10^4^, which is experimentally determined, the oocyte was thought to appear in the ROI. Then, an appropriate constant aspiration pressure (10 kPa for porcine oocyte) was experimentally determined and exerted in the MM to hold the target oocyte. The cytoplasm contour of the target oocyte and other cells were detected to locate them [33]. If the distance between their centers was smaller than a threshold value determined by the size of the operated cell (160 um for pig oocytes), the adhesion between the target cell and the other cell was determined. Then an aspiration pressure, calculated in the next section, was exerted in the FM to pull other cells back and break their adhesion to the held target cell.

#### 2.1.3. Aspiration Pressure to Break Cell Adhesion

**Figure 7:**
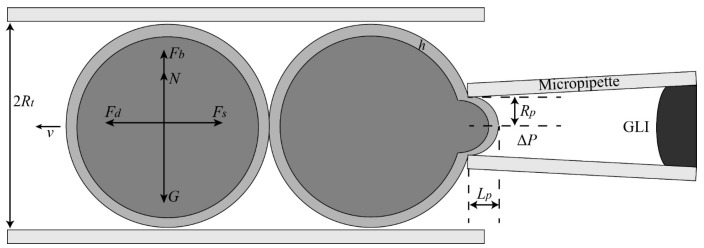
Schematic figure of force analysis when drawing back the other cell from the held cell.

As the oocytes stored in the feeder micropipette (FM) were usually adhered to each other, the pressure to draw back the adhesive oocytes was calculated using force analysis on cells (as shown in Figure 7).

When the cells are in the balanced state, we have

(3) $$G=N+F_{b}$$

(4) $$F_{d}=F_{s}$$

where *G* is the gravity, *N* is the branching force, *F_b_* is the buoyancy force, *F_s_* is the stick force between the adhesive oocytes, and *F_d_* is the dragging force generated by the fluidic flow.

The dragging force *F_d_* caused by the fluidic flow can be calculated as [34]

(5) $$F_{d}=\frac{1}{2}\rho_{L}v^{2}C_{D}S=\frac{1}{2}\rho_{L}v^{2}C_{D}\pi R^{2}$$

where $\rho_{L}$ is the density of the liquid, *v* is the average velocity of the fluid near the oocyte, *C_D_* is the drag coefficient of an oocyte, *S* is the cross-sectional area of the FM, and *R* is the radius of the FM.

According to Hagen–Poiseuille, we have

(6) $$Q=\frac{\pi d^{4}\Delta P}{128\mu l}$$

(7) $$v=\frac{R^{2}\Delta P}{8\mu l}$$

where *Q* is the volumetric flow rate, *d* is the diameter of the FM, Δ*P* is the pressure difference between the tip and the bending place of FM, *μ* is the viscosity coefficient of the liquid, and *l* is the length of FM.

Substituting Equations (4) and (5) into Equation (7), we have the relationship between Δ*P* and *F_s_*:

(8) $$\Delta P=\frac{8\mu l}{R^{3}}\sqrt{\frac{2F_{s}}{\rho_{L}C_{D}\pi }}$$

According to Equation (8), to calculate required aspiration pressure to break the cell adhesion, we need to calibrate the stick force *F_s_* between cells by experiments. In this experiment, the oocyte on the right was held by a large enough aspiration pressure, and the oocyte on the left was gently held with a negative pressure Δ*P_C_*. We gently increased this pressure until the oocytes could be separated with this pressure (Video S2 and Video S3 show the experiment where the adhesive oocytes could not and could be separated).

**Figure 8:**
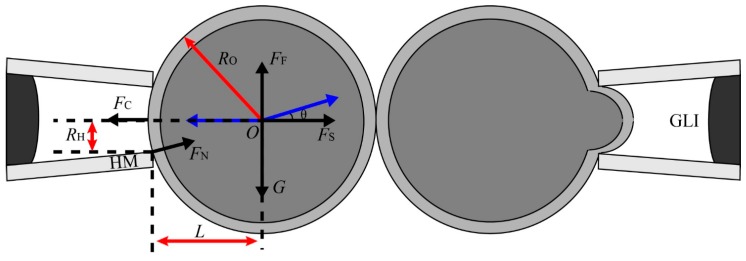
Diagram of force analysis in the calibration experiment.

Firstly, the force analysis is carried out for the calibration experiment (as shown in Figure 8).

The oocyte aspirated by Δ*P_C_* is an equilibrium state, and the oocyte under this state can be treated as a rigid body, according to the static theory [35]. Then, we have

(9) $$F_{C}=F_{S}+F_{N}\cos\theta$$

(10) $$F_{F}+F_{N}\sin\theta =G$$

(11) $$F_{S}=F_{C}-\left(G-F_{F}\right)\cot\theta$$

where *F_C_* is the force caused by the negative pressure Δ*P_C_*, *F_S_* is the stick force between the two adhesive oocytes, *F_N_* is the contacting force of the holding micropipette (HM) on the oocyte, *θ* is the angle between *F_N_* and *F_S_*, *F_F_* is the buoyancy force, and *G* is the gravity.

*F_C_*, *F_F_* and *G* can be calculated as,

(12) $$F_{C}=\pi R_{H}{}^{2}\Delta P_{C}$$

(13) $$F_{F}=\frac{4}{3}\pi R_{O}{}^{3}\rho_{L}g$$

(14) $$G=mg=\frac{4}{3}\pi R_{O}{}^{3}\rho_{O}g$$

respectively, where *R_H_* is the radius of the HM’s opening, Δ*P_C_* is the calibration negative pressure, *R_O_* is radius of oocyte, *g* is the gravitational acceleration, *m* is the mass of the oocyte, and $\rho_{O}$ is the density of the oocyte.

According to our previous research, the negative pressure Δ*P_C_* is generated by reducing the initial pressure *P_ic_* to *P_ic_*–Δ*P_i_* [7],

(15) $$\Delta P_{C}=\Delta P_{i}+2\mu \cos\alpha^{\prime }/R^{\prime }-P_{ic}$$

where α′ and *R*′ are the values of the contacting angle and inner radius of the micropipette at the gas-liquid interface (GLI).

Substituting Equations (12), (13), and (14) into Equation (11), we have

(16) $$F_{S}=\pi R_{H}{}^{2}\Delta P_{C}-\frac{4}{3}\pi R_{O}{}^{3}g\left(\rho_{O}-\rho_{L}\right)\cot\theta$$

where cot*θ* can be calculated as

(17) $$\cot\theta =\frac{L}{R_{H}}$$

where *L* is the distance between the center of oocyte *O* and the HM opening, which can be calculated as:

(18) $$L=\sqrt{R_{O}{}^{2}-R_{H}{}^{2}}$$

Then, substituting Equation (16) into Equation (8), we could get the required drawing back pressure Δ*P*:

(19) $$\Delta P=\frac{8\mu l}{R^{3}}\sqrt{\frac{2}{\rho_{L}C_{D}}\left(R_{H}{}^{2}\Delta P_{C}-\frac{4}{3}R_{O}{}^{3}g\left(\rho_{O}-\rho_{L}\right)\cot\theta \right)}$$

#### 2.1.4. Automated Aspiration of Cell

**Figure 9:**
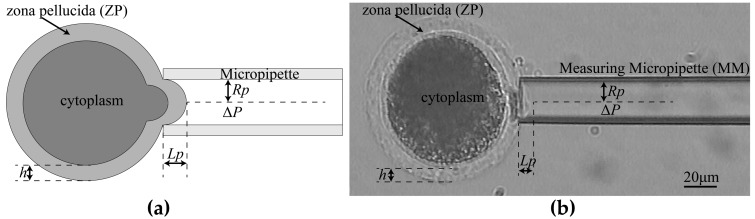
Automated aspiration of the oocyte. (**a**) Diagram of the shell model using to estimate the Young’s modulus of the zona pellucida of the oocyte. (**b**) The MA of porcine oocytes.

We use the commonly used shell model [36,37] to estimate the Young’s modulus of the zona pellucid (ZP) of oocyte (as shown in Figure 9a), and the Young’s modulus of oocyte can be estimated by

(20) $$E=2C(h^{*})(1-\nu^{2})\left(\frac{\Delta P}{\Delta L/R_{P}}\right)$$

where *v*, assuming incompressibility (*v* = 0.5), is evaluated; h* represents oocyte’s dimensionless thickness, which is defined as the ratio of the ZP’s thickness (*h*) and the micropipette’s radius (*R_p_*); Δ*P* is the suction pressure; Δ*L* is the increment with the changing of the aspiration pressure; C(h*) is a function of h* and can be estimated by the following equation,

(21) $$C(h^{*})=\left\{ \begin{array}{cc} \frac{a+c\ln(h^{*})+e\ln^{2}(h^{*})+g\ln^{3}(h^{*})+i\ln^{4}(h^{*})}{1+b\ln(h^{*})+d\ln^{2}(h^{*})+f\ln^{3}(h^{*})+h\ln^{4}(h^{*})+j\ln^{5}(h^{*})} & 0.1\leq h^{*}\leq 50\\ 0.64395655 & h^{*}\geq 50 \end{array}\right.$$

where *a* = 1.070275412, *b* = 0.592405186, *c* = −0.44373788, *d* = 0.126723221, *e* = 0.721290633, *f* = 0.074985305, *g* = −0.14390482, *h* = 0.027220129, *i* = 0.040156098, *j* = 0.00132358.

As the oocyte has been held by the MM in Section 2.1.2, we started to measure its Young’s modulus immediately after the MM retreat out of the FM. In this step, a series of steep decreases of aspiration pressure were exerted on the cell according to our previously developed balanced pressure model, until the whole cell flowed into the MM (as shown in Figure 9b).

#### 2.1.5. Oocytes Preparation

Ovaries were collected at a local slaughterhouse and transported to the laboratory in a thermos flask with 35–37 °C sterilized physiological saline within 2 h. Ovaries were then washed twice with 37 °C sterilized physiological saline containing 100 IU/L penicillin and 50 mg/L streptomycin. Oocytes were aspirated from follicles (2–6 mm in diameter) with an 18-guage needle attached to a disposable 10 mL syringe. After being washed three times with TL-HEPES-PVA, the oocytes with uniform ooplasm and compact cumulus cells (COCs) were maturation cultured in vitro for 42 h with 4 different treatments. Group A: COCs were cultured for 42 h in the maturation medium supplemented with 1 ng/mL FSH and LH. Group B: COCs were cultured for 20 h in the maturation medium supplemented with 1 ng/mL FSH and LH and then were cultured for another 22 h in the medium without FSH and LH. Group C: COCs were cultured for 42 h in the maturation medium supplemented with 10 ng/mL FSH and LH. Group D: COCs were cultured for 20 h in the maturation medium supplemented with 10 ng/mL FSH and LH and then were cultured for another 22 h in the medium without FSH and LH. All the groups were cultured in a 39 °C humidified incubator containing 5% CO_2_ in air.

Oocytes in the four groups were denuded respectively by gentle pipetting in 0.1% hyaluronidase after IVM. After washing three times with M199 (Earle’s Salt with 25 mM HEPES buffer), the denuded oocytes were used for elastic detection using MA.

### 2.2. Robotic MA Process for Batch Cells

**Figure 10:**
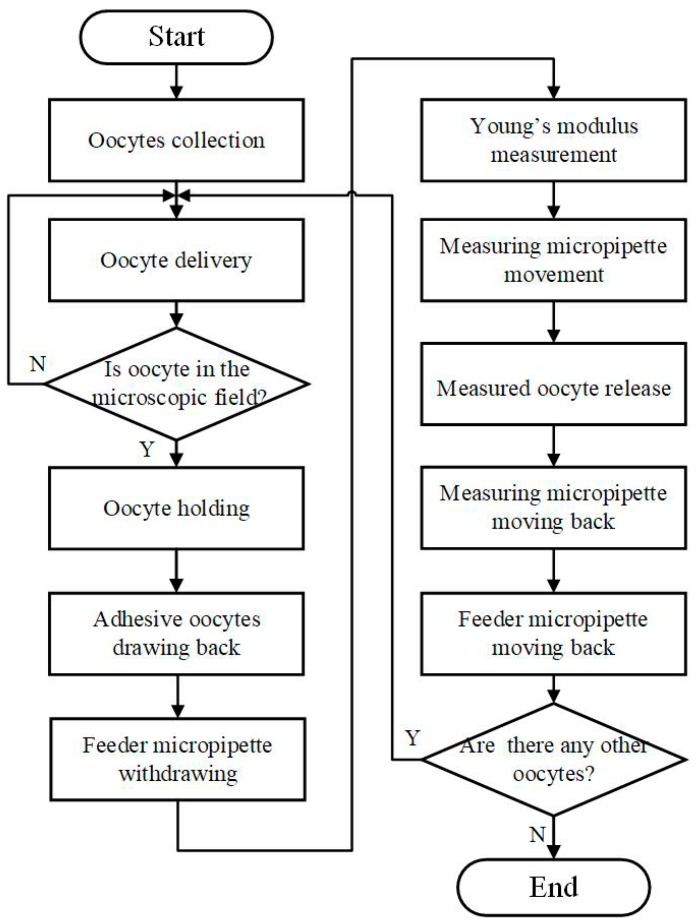
The operation flow of new MA process for batch cells.

Based on above work, we designed a new automated MA procedure for operating multiple cells one by one, as shown in Figure 10 (see Video S4 for the new batch measuring process). The detailed steps are listed as follows:

1. Collect multiple oocytes into the FM.
2. Insert MM into the FM automatically.
3. Apply positive pressure in the FM until the oocyte is delivered to the microscopic field.
4. Hold the oocyte with negative pressure in the MM.
5. Draw back the adhesive oocytes with the calculated negative pressure in the FM and withdraw the FM.
6. Measure the Young’s modulus of ZP by MM.
7. Move MM to the “Measured Area”. Release the measured oocyte with positive pressure in MM.
8. Move the MM back and move the FM back.
9. Repeat steps 2–7 if there are oocytes to be measured, otherwise end the measurement process.

### 2.3. Statistical Analysis

Data were evaluated by one-way analysis of variance (ANOVA) with Tukey test for comparisons between groups using IBM SPSS Statistics 20 and were expressed as mean ± SEM. A *p* value less than 0.05 was considered as significant difference.

### 2.4. Ethical Statement

All the procedures were approved by the Animal Care and Use Committee of Tianjin Animal Science and Veterinary Research Institute and were performed in accordance with the NIH Guide for the Care and Use of Laboratory Animals (No. 8023, revised in 1996).

## 3. Results

### 3.1. System Setup

**Figure 11:**
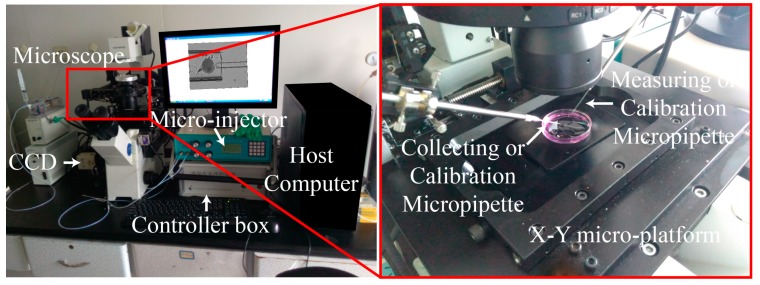
NK-MR601 micro-operation system.

The experiment in this paper was performed on the self-developed NK-MR601 micro-operation system [32,35,38,39] (as shown in Figure 11). The system consists of an optical microscope (CK-40, Olympus, Tokyo, Japan); a CCD camera (W-V-460, Panasonic, Tokyo, Japan) for the acquisition of the real-time image at 20 frame/s; a motorized X-Y stage (with a travel range of 100 mm with a repeatability of ±1 μm/s and a maximum speed of 2 mm/s); a pair of XYZ manipulators (travel range of 50 mm with a repeatability of ±1 μm/s and a maximum speed of 1 mm/s); an in-house developed micro-injector provides the negative and positive pressure; and an in-house developed motion control box controlling the motion of micro-platform, micro-manipulators, and micro-injector through the host computer.

The feeder micropipette (FM), the measuring micropipette (MM) and the micropipettes for calibration were all made from borosilicate glass tubes with an outer diameter of 1 mm and an inner diameter of 0.8 mm. The micropipettes were all pulled by the puller (MODEL P-97, Sutter Instrument) and fractured by the microforge (MF-900, NARISHIGE, Tokyo, Japan). The inner diameter of the FM was 180–200 μm. The inner diameter of the MM and micropipettes for calibration was 40–80 μm.

### 3.2. Drawing Back Pressure Calculation

According to our previous study, we know the density of the culture media was approximately 1008.2 kg/m^3^ [35] and the density of the oocyte was about 1150.6 ± 39.2 kg/m^3^ [35], which was set as 1150.6 kg/m^3^ in this paper. The viscosity coefficient of the liquid was about 79.30 ± 0.63 mN/m [7]. The gravitational acceleration was set as 9.8 N/kg. We measured the contacting angle α’ and the radius of the HM’s opening *R_H_*. In this paper, α′ is about 25°, and *R_H_* is about 14.375 μm. Then, in the calibration experiments, the stick force is about (4.74 ± 0.09) × 10^−^^6^ N.

The dragging coefficient of a sphere oocyte is equal to 0.47 [34]. In this paper, the radius of the opening of the FM is 95.3 μm and the length of the FM is 1597.043 μm. Then, the needed drawing back pressure Δ*P* can be calculated by Equation (19): 9181.03–9506.91 Pa. In the experiments, this pressure was set as 9600 Pa (a little bigger than the calculated results) to ensure the success of separating oocytes. Using this aspiration pressure, the success rate to break the adhesion between cells is 100% (*n* = 100).

### 3.3. Young’s Modulus Detection Results

**Figure 12:**
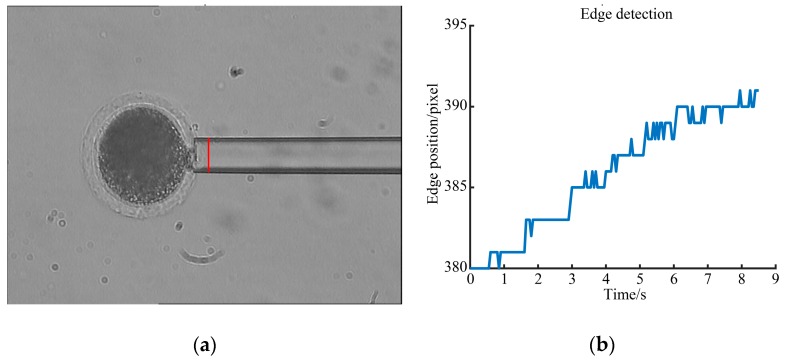
Oocyte aspiration. (**a**) Oocyte aspiration for measuring its Young’s modulus; (**b**) Oocyte edge position in the measuring process.

The measuring micropipette (MM) starts to aspirate, meanwhile, the elongation of ZP in the micropipette is calculated according to literature [7], and the relationship between aspiration pressure and extending length is obtained (as shown in Figure 12, see Video S5 for the oocyte deformation and edge detection process).

**Figure 13:**
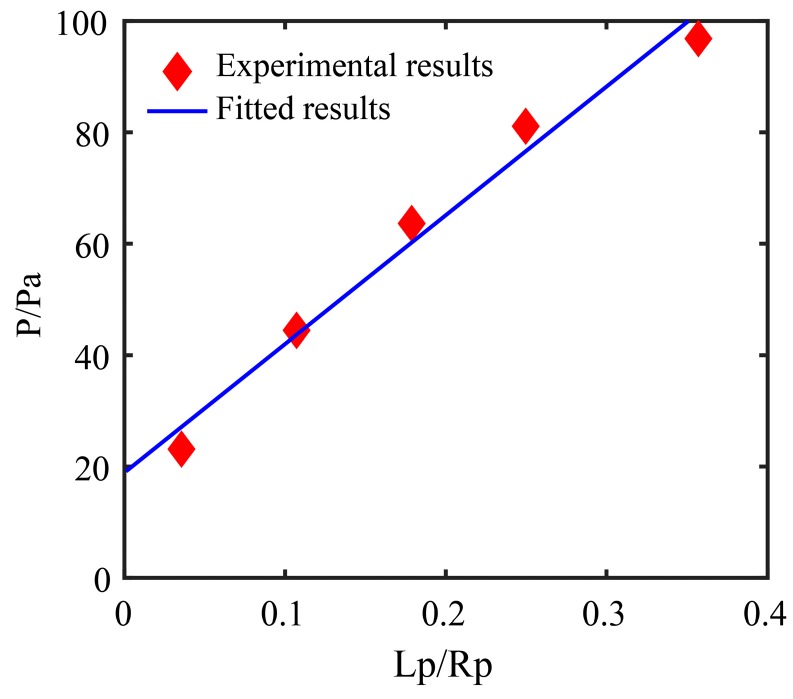
The relationship between the aspiration pressure and the elongation of the zona pellucida (ZP) when its Young’s modulus is detected as 22.80 kPa.

Then, the Young’s modules of ZP is obtained according to the slope of the fitted curve and Equation (19). Figure 13 shows the relationship between the aspiration pressure and the elongation of the ZP when its Young’s modulus is detected as 22.80 kPa.

To verify the validity of the new method, we compared the ZP Young’s modulus measured with the traditional measuring method and the new batch measuring method. The oocytes measured by the two methods were cultured in the same environment. The Young’s modulus measured by the two methods shows no significant difference (*p* > 0.05) (as shown in Table 1).

Table 2 shows measurement speed comparison results between the traditional MA method and our method (*n* = 22). We could find that the new method saves much time in the “Localization” and “Hold” section. In the “Localization” section, the traditional method needs to search for oocytes scattered in a relatively large area in the Petri dish, while the new method only needs to deliver the oocytes in the FM to the microscopic field, saving searching time for the next oocyte. In the “Hold” section, in the new method, the target oocyte was caught by the MM and the adhesive oocytes were then separated by the negative pressure in the FM. In comparison, in the traditional method, if the target oocyte was adhered by other oocytes, they needed to release the oocyte and search for another oocyte. Our system succeeded to operate 22 cells continuously. The new batch method spends 0.5 min on average for measuring one cell, while the traditional cell measurement takes 2 min. The measurement speed of the new batch measuring method is quadrupled in comparison to that of the traditional measuring method.

To demonstrate the necessity of accelerating average cell operation speed in MA, we compared the Young’s modulus of the oocytes that were cultured for 42 h, which are considered as matured, and the oocytes that were aged (six hours after maturation) according to literature [40]. Table 3 shows the results of the Young’s modulus of the above two group of oocytes. The average ZP Young’s modulus of the aged oocytes is significantly larger than that of mature oocytes (*p* < 0.05). The ZP Young’s modulus increased more than 1 kPa on average per hour according to the results in Table 3. The hardening process of the ZP during oocyte aging might be due to the cortical granule disappearance [41]. In the traditional measuring process, the cells’ Young’s modulus varied resulting from cell aging during long waiting time of other cells. For example, if we measured the Young’s modulus of 20 oocytes, the oocytes in the traditional method would have to wait for another 30 min compared with the new robotic measurement method, which may cause about a 500 Pa increase for ZP Young’s modulus according to the obtained average ZP harden speed in Table 3. Thus, the Young’s modulus measured by our method, which has a faster method, is more reliable.

### 3.4. Oocyte Elasticity under Different Culture Environments

We measured the Young’s modulus of the oocytes cultured in four different environments as mentioned in Section 2.1.5, “Oocytes Preparation”. By using this method, we compared the ZP Young’s Modulus of the oocytes in different cultured conditions to find an optimized culture environment for biological applications. Table 4 shows the oocytes’ Young’s modulus in the four different culture environments and demonstrates that the oocyte Young’s modulus in Group C^(a)^ is significantly bigger than that in Groups A^(b)^, B^(b)^ and D^(b)^ (*p* < 0.05). That is, the oocytes cultured in environment C are stiffer, and the oocytes cultured in environments A, B and D are more resilient. We concluded that oocytes cultured under group C conditions matured earlier. The oocytes in Group D matured slightly earlier than those in Group A and the oocytes in Group A matured slightly earlier than those Group B.

## 4. Conclusions

In this paper, we provided a robotic micropipette aspiration (MA) system for batch cell Young’s modulus measurement. Firstly, we introduced a feeder micropipette (FM) to store and transport the cells and a measurement micropipette (MM) to pick up and measure the cells one-by-one automatically. We determined the maximum permissible tilt angle of MM and FM to ensure that the MM can insert into the FM was calculated (17°). We detected the adhesion between the target cell and the other cells and we calculated the required aspiration pressure to break the adhesion between the adhesive cells (9600 Pa). Secondly, we measured the Young’s modulus of the porcine oocytes by the new batch method and traditional method. The experimental result shows that our system was reliable, and the measuring speed was quadrupled in comparison to existing methods (2 min/cell vs. 0.5 min/cell). The MA results demonstrated that our system was able to continuously operate more than 20 cells. Thirdly, we measured the Young’s modulus of the porcine oocytes cultured in different environments by this new batch method. The experimental results showed that the zona pellucida (ZP) Young’s modulus distribution of pig ovum cultured in four different environmental conditions was good enough to find better culturing protocols for cell maturing.

## Figures and Tables

**Table 1:** Measuring results comparison between traditional and new method.

Groups	Amount	Elasticity (kPa)
New	15	19.41 ± 4.33
Traditional	15	19.20 ± 5.43

**Table 2:** Measuring speed comparison between traditional and new method.

Groups	Localization	Hold	Measure	Release	Total
New method	4 s	4 s	11 s	11 s	0.5 min
Traditional method	58 s	40 s	11 s	11 s	2 min

**Table 3:** Comparison between mature oocytes and aged oocytes.

Groups	Amount	Elasticity (kPa)
Mature oocytes	15	10.26 ± 5.65
Aged oocytes	15	18.51 ± 3.41

**Table 4:** The Young’s modulus of the oocytes from four groups.

Groups	Amount	Elasticity (kPa)
A	15	8.15 ± 4.93
B	15	5.55 ± 3.02
C	10	22.55 ± 10.65
D	12	9.45 ± 4.83

## Supplementary Materials

The following are available online at https://www.mdpi.com/2072-666X/10/5/348/s1, Video S1: The oocyte detection during the delivery process. Video S2 and Video S3 show the experiment that the adhesive oocytes could not and could be separated. Video S4: The new batch measuring process. Video S5: The oocyte deformation and edge detection process.
